# Similar Sensitivity of SARS-CoV-2 Detection in Oropharyngeal/Nasopharyngeal and Saliva Samples on the Hologic Panther Platform

**DOI:** 10.3390/diagnostics13030347

**Published:** 2023-01-18

**Authors:** Ali Vahidnia, Dennis Souverein, Sjoerd M. Euser, Milly Haverkort, Elise Noordhuis, Thomas Z. I. van Zijl, Jayant Kalpoe, Jan C. Sinnige, Bjorn L. Herpers

**Affiliations:** 1 Regional Public Health Laboratory Kennemerland, 2035 RC Haarlem, The Netherlands; 2 Public Health Service Kennemerland, 2015 CK Haarlem, The Netherlands

**Keywords:** saliva, TMA, SARS-CoV-2, Hologic, sensitivity

## Abstract

Background: Oropharyngeal (OP) and nasopharyngeal (NP) sampling has historically been considered the reference specimen type used for respiratory virus detection. Saliva could be a less invasive alternative for SARS-CoV-2 detection, but limited evidence is available. Methods: The technical and clinical performance of saliva was compared to OP/NP on the Hologic Panther platform with two Aptima assays, the End-Point Transcription-Mediated Amplification assay (EP-TMA) and Real-Time Transcription-Mediated Amplification assay (RT-TMA). The samples were collected at the Public Health Service Testing Site XL location in Schiphol Amsterdam Airport. At the site, the Regional Public Health Laboratory Kennemerland (RPHLK) has a fully equipped laboratory facility. Results: A total of 374 samples (187 OP/NP swabs and 187 saliva samples) were collected from 187 unique patients. The Real-Time Transcription-Mediated Amplification assay (RT-TMA) resulted in comparable sensitivities for the detection of SARS-CoV-2 in both the OP/NP swabs (88.3%; 113/128) and saliva samples (87.5%; 112/128). The End-Point Transcription-Mediated Amplification assay (EP-TMA) analyses showed a similar sensitivity (86.7%; 111/128) in the OP/NP swabs but a lower sensitivity in the saliva samples (80.5%; 103/128). Within the discordant analyses, we found no associations in the symptoms, earlier SARS-CoV-2 infections and eating, smoking, drinking and tooth brushing habits within one hour before testing. Conclusions: The Hologic Panther platform Real-Time Transcription-Mediated Amplification assay (RT-TMA) yields a sensitivity for the detection of SARS-CoV-2 in saliva that is comparable to the OP/NP swabs derived from participants presenting themselves at a public health testing facility with minimal or mild symptoms.

## 1. Introduction

Currently, most SARS-CoV-2-infected individuals are identified by a successful Nucleic Acid Amplification Test (NAAT) of the virus from oropharyngeal (OP) and/or nasopharyngeal (NP) swabs [1,2]. The identification of infected individuals using high-throughput assays for SARS-CoV-2 is a crucial way to prevent the spread of the disease. Since the large-scale (self)testing of symptomatic and asymptomatic individuals has become a reality, innovation in sampling is as important as innovation in testing to increase both the testing comfort for individuals and the test validity. Although several studies analyzed alternative sampling methods such as mid-turbinate and saliva sampling, OP/NP sampling has historically been considered the reference specimen type used for respiratory virus detection and is therefore predominantly implemented [3]. On the other hand, OP/NP sampling is experienced as uncomfortable and might lead to limiting the willingness to come forward for testing, especially in asymptomatic individuals [4]. For these reasons, many laboratories have explored different sampling techniques and ease-of-use sampling methods, such as saliva [5,6].

Several review studies have recently demonstrated that saliva NAAT diagnostic accuracy is similar to that of an NAAT nasopharyngeal swab, making saliva a clinically acceptable alternative specimen collection method [7,8]. In addition, Lee et al. published a large review and meta-analysis of the clinical performance of saliva against several competitors [2]. Across the 25 included studies that compared saliva against all positives including NP (or OP/NP), saliva yielded an 88% positive agreement and NP a 94% positive agreement, although the meta-analysis also showed that a large heterogeneity existed between the studies (with an I^2^ higher than 85%). The authors attributed this high heterogeneity to differences in the sample procedures, population characteristics and testing procedures.

Only one of the included studies utilized transcription-mediated amplification (TMA) as the testing procedure [9]. The percentage positive agreement was almost identical for the saliva (94%) and NP (93%) against all positives. Unfortunately, this study lacked information on the inclusion of symptom duration, earlier SARS-CoV-2 infections, symptoms and possible confounders, such as eating, drinking and tooth brushing, which limited the interpretation of the differences between the two sampling methods.

COVID-19 (SARS-CoV-2) and seasonal flu (influenza viruses) are both infectious respiratory illnesses but are caused by different viruses. Because some of the symptoms of COVID-19 and seasonal flu are similar, it may be hard to tell the difference between them based on symptoms alone [10]. In the current time period in which infection control measures are more strict for SARS-CoV-2 than influenza, it is therefore important to make a distinction between both.

The goal of this study was to study the clinical performance of saliva compared to OP/NP on the Hologic Panther platform with two Aptima assays, the End-Point Transcription-Mediated Amplification assay (EP-TMA) which produces only results for SARS-CoV-2 and a newly introduced Real-Time Transcription-Mediated Amplification assay (RT-TMA) which also produces results for influenza virus A and B in addition to SARS-CoV-2.

## 2. Methods

### 2.1. Ethics Statement

The Medical Ethical Committee of the Utrecht UMC approved this study on 21 January 2022 (Study number:22/058). The data were anonymized after collection and analyzed under code.

### 2.2. Setting, Study Design and Participants

The study was designed as a clinical validation study at the Public Health Service Testing Site XL location in Schiphol Amsterdam Airport where testing was performed by appointment only. At the site, the Regional Public Health Laboratory Kennemerland (RPHLK) has a fully equipped laboratory facility. At the entrance of the testing site, persons > 12 years of age were approached for the study participation and asked to give written informed consent. After inclusion, they were enrolled in the study and directed to one of the dedicated testing posts for sampling. The participants were asked to fill out a short questionnaire including questions on symptoms, earlier SARS-CoV-2 infections, eating, smoking, drinking and tooth brushing habits within one hour before testing. The study was conducted for two consecutive days.

### 2.3. Sample Collection and Processing

After filling out the questionnaire, OP/NP and saliva samples were collected consecutively. The OP/NP swab was collected by trained personal following a uniform sampling method. Saliva was collected by spitting into a clean container and diluted by pipetting 0.5 mL of saliva with 2 mL of PCR-grade water. From the diluted saliva sample, 0.5 mL was pipetted into a Specimen Lysis Tube from Hologic (Hologic, Inc., San Diego, CA, USA).

### 2.4. SARS-CoV-2 Detection Using the Aptima EP-TMA SARS-CoV-2 Assay

The Aptima SARS-CoV-2 assay on the Panther instrument (Hologic, Inc., San Diego, CA, USA) targets two parts of the ORF1 ab region of the SARS-CoV-2 genome and one internal control. This test is based on End-Point Transcription-Mediated Amplification (EP-TMA), which is a binary test for the presence or absence of SARS-CoV-2 [11,12]. After amplification, chemiluminescent probes hybridize to amplicons and emit light measured by a luminometer in relative light units (RLUs). For valid samples, the RLU value will range between approximately 250 (internal control only) and 1250 RLUs with a positive internal control, and two positive targets (of 500 RLUs each). Assay results are determined by a cutoff based on the total RLU and the kinetic curve type.

### 2.5. SARS-CoV-2 Detection Using the Aptima RT-TMA SARS-CoV-2/Flu Assay

Second, a newly introduced test on the same platform was performed, a quantitative test based on Real-Time Transcription-Mediated Amplification (RT-TMA) indicative of viral load by generating time to positivity (TTP). The Aptima SARS-CoV-2/Flu assay combines the technologies of target capture, RT-TMA and real-time detection of amplicons using fluorescently labeled torches. When the Aptima SARS-CoV-2/Flu assay is performed on the Panther system, an Internal Control (IC) nucleic acid is added to each specimen reaction, and the IC along with the target RNA molecules are isolated from specimens by use of capture-specific oligomers via target capture that utilizes magnetic microparticles. After the target capture steps are completed, the specimens are ready for amplification. In addition to SARS-CoV-2, also the presence of influenza A and B is measured by the RT-TMA kit. The Aptima SARS-CoV-2/Flu assay amplifies and detects two conserved regions of the ORF1 ab gene in the same reaction for SARS-CoV-2 (FAM fluorescent channel), one region of the Matrix gene for Flu A (ROX fluorescent channel) and one region of the Matrix gene for Flu B (HEX fluorescent channel). The two regions of the SARS-CoV-2 target are not differentiated, and amplification of either or both regions leads to relative fluorescence units (RFU) signal. The assay results for all targets are determined by fluorescence and emergence cutoffs (Aptima SARS-CoV-2/Flu-Panther System, AW-22365-001 Rev. 003). To generate valid results, a set of assay controls must be tested. One replicate of the negative assay control and positive assay control must be tested with each run at an administrator-specified interval of up to 24 h. During processing, criteria for acceptance of the assay controls are automatically verified by the Panther system. To generate valid results, the assay controls must pass a series of validity checks performed by the Panther system.

### 2.6. Statistical Analysis

Test results for the EP- and RT-TMA assay with OP/NP were compared to their reciprocal saliva samples and presented by calculating sensitivity and specificity using 2 × 2 contingency tables. The 95% confidence intervals were calculated with the Wilson score confidence interval. Discordant results were further analyzed by searching for associations in the questionnaires patient characteristics, symptoms, earlier infections and habits one hour before testing using logistic regression analysis. All statistical analyses were performed with R and RStudio (R version 4.0.3).

## 3. Results

On the 9 and 10 May 2022, 374 samples (187 OP/NP swabs and 187 saliva samples) were collected from 187 unique patients. With respect to the study population, similar age distributions and SARS-CoV-2 prevalence were seen compared to the routinely tested population from the public health service testing facilities tested on the same two days (Table 1).

The baseline characteristics showed that a higher age was significantly associated with a positive SARS-CoV-2 TMA (testing positive for one of the four TMA tests) and that an earlier infection was associated with a negative SARS-CoV-2 TMA (Table 2). From the 187 included persons, some had invalid testing results: 0 tests (0%) for the EP-TMA OP/NP, 6 tests (3.2%) for the EP-TMA saliva, 2 tests (1.1%) for the RT-TMA OP/NP, and 17 tests (9.1%) for the RT-TMA saliva.

### 3.1. Clinical Validation Results

**Figure 1:**
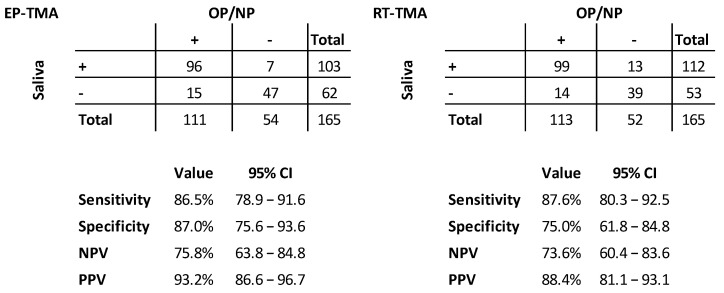
Test results and characteristics for EP-TMA (left) and RT-TMA (right) comparing saliva samples with OP/NP swabs (*n* = 165). EP-TMA = End-Point Transcription-Mediated Amplification; RT-TMA = Real-Time Transcription-Mediated Amplification; CI = confidence interval; NPV = negative predictive value; PPV = positive predictive value.

**Figure 2:**
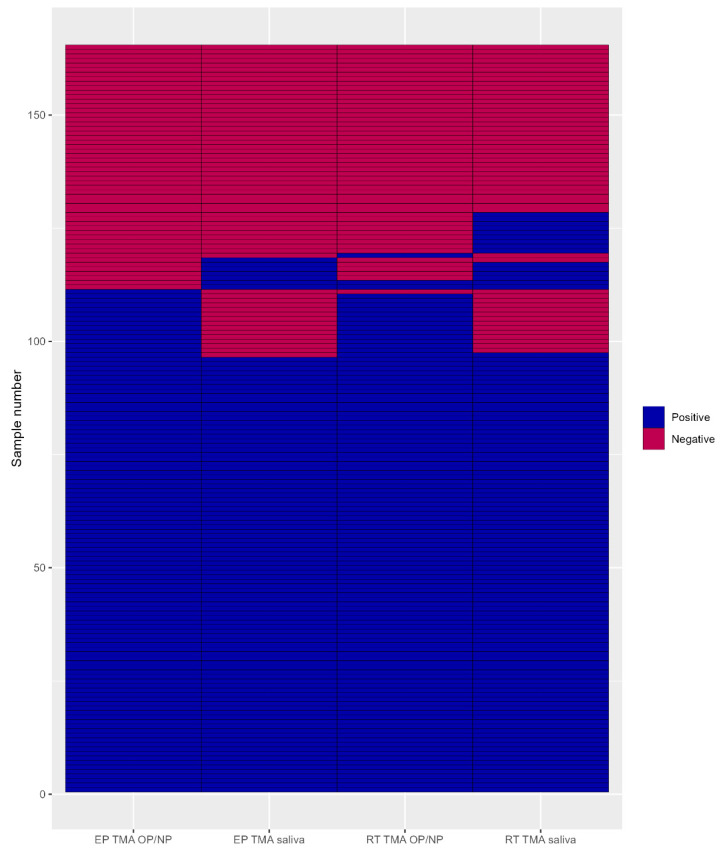
Test results for EP-TMA and RT-TMA (*n* = 165). EP-TMA = End-Point Transcription-Mediated Amplification; RT-TMA= Real-Time Transcription-Mediated Amplification.

For the clinical validation analyses, the invalid results were excluded, resulting in 165 (88.2%) persons who had four valid TMA results available. Figure 1 shows the clinical performance parameters of the comparison between the saliva and OP/NP (reference) within their specific assay groups. Similar sensitivities were found for the EP-TMA (86.5%) and RT-TMA (87.6%). The comparison of the specificities of both assays showed a lower specificity for the RT-TMA (75.0%) compared to the EP-TMA (87.0%), reflecting more saliva positives and OP/NP negatives for the RT-TMA assay. When a combined golden standard was composed (with ‘true positives’ defined as participants with one or more of the four tests positive), the following sensitivities were found for the four assay/specimen combinations in descending order: RT-TMA OP/NP (88.3%; 113/128), RT-TMA saliva (87.5%; 112/128), EP-TMA OP/NP (86.7%; 111/128) and EP-TMA saliva (80.5%; 103/128). The results of all four tests are shown in Figure 2.

### 3.2. Discordant Analysis

The discordant results between the EP-TMA specimens were further analyzed with respect to the data collected using the questionnaire at the sample collection. Additionally, for the RT-TMA assay, the time to positivity was compared between the samples. For the EP-TMA, 15 persons tested positive on the OP/NP swab and negative in saliva. Within the questionnaire data, no symptoms and/or habits before testing could be attributed to these discrepancies. Moreover, for the EP-TMA, seven persons tested saliva positive and OP/NP negative. From the questionnaire data, several trends could be identified. It seemed, for example, that these persons had reported more earlier infections (42.9 vs. 15.0%; OR: 4.25 [0.77–21.23]) and had experienced less symptoms (sore throat, cough, myalgia, shortness of breath and fever) at the moment of testing, although no significant differences were found. For the RT-TMA, 14 persons tested positive on the OP/NP swab and negative in saliva. In line with the EP-TMA results, no associations could be found within the questionnaire data. Moreover, for the RT-TMA, 13 persons tested positive in saliva and OP/NP negative. In line with the EP-TMA analyses, these persons also seemed to have reported less symptoms, although only coughing was reported significantly less often (15.4 vs. 51.5%; OR: 0.17 [0.03–0.68]).

**Figure 3:**
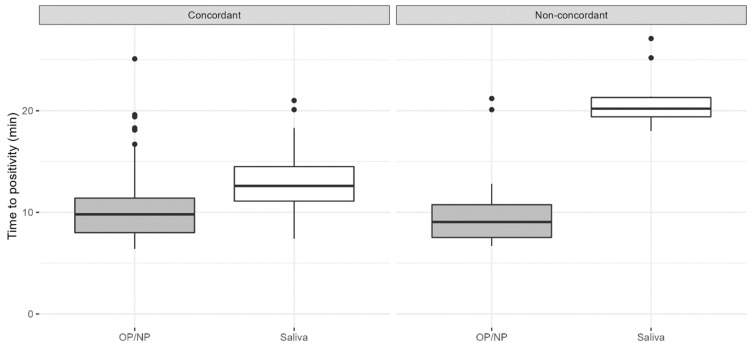
Time to positivity (TTP) of the RT-TMA compared between OP/NP swabs and saliva samples for concordant (both positive) samples (left panel), OP/NP positive and saliva negative samples and OP/NP negative and saliva positive samples (right panel). These box-whisker-plots present the median (IQR) time to positivity for the positive samples (OP/NP and/or saliva) from participants with concordant RT-TMA results (both OP/NP swab and saliva sample positive) and non-concordant RT-TMA results (either OP/NP swab or saliva samples negative).

Comparing the time to positivity (TTP) within the RT-TMA, the discordant results showed a higher TTP within the group of saliva positive with OP/NP negative samples, which may indicate a lower viral load of these samples. On the other hand, the TTP within the OP/NP positive/saliva negative group showed a similar TTP compared to the concordant samples (Figure 3).

### 3.3. Influenza Testing Results with RT-TMA

The RT-TMA assay also generates results for Influenza A and B. In 165 OP/NP swabs, we found 12 positive samples (7.3%) for Influenza A. For saliva, we found eight concordant positives, resulting in a sensitivity of 66.7% (8/12). All the saliva negatives were also negative with the OP/NP, resulting in a 100% specificity (153/153). All the persons tested negative for Influenza B. Three co-infections (1.8%) with influenza and SARS-CoV-2 were found.

## 4. Discussion

Based on a combined golden standard (with ‘true positives’ defined as participants with one or more of the four tests positive), this study showed that the Real-Time Transcription-Mediated Amplification assay (RT-TMA) resulted in comparable sensitivities for the detection of SARS-CoV-2 in both the OP/NP swabs (88.3%; 113/128) and saliva samples (87.5%; 112/128) in participants presenting themselves for testing in a public health service testing facility. The End-Point Transcription-Mediated Amplification assay (EP-TMA) analyses showed a similar sensitivity (86.7%; 111/128) in the OP/NP swabs but a lower sensitivity in the saliva samples (80.5%; 103/128). These results are in line with previous studies where the OP/NP and saliva collection methods were compared with respect to their diagnostic accuracy in both children and adults [2,7,8,13]. One earlier study compared the saliva against the NP for the EP-TMA assay and found sensitivities of 94% for saliva and 93% for the NP against all positives [9]. For the RT-TMA, no earlier studies were found for either saliva or other sample types. For the EP-TMA assay, several earlier studies were performed comparing the OP/NP against several PCR assays [14,15]. These studies showed comparable and sometimes higher sensitivities for the EP-TMA assay, dependent on the comparator PCR assay.

The Hologic Panther platform is an automated complete sample-to-result instrument for testing up to 1200 samples per day per instrument. During the height of the pandemic, having fully automated systems with high-throughput capacity testing with minimum hands-on time was necessary. During the pandemic and this study, we worked alongside our partner, the Public Health Service Kennemerland, facilitating SARS-CoV-2 testing sites. Our facility housed eight Hologic Panther platforms for SARS-CoV-2 screening, enabling us to screen 10,000 persons on a daily basis. For the duration of this study, one of these platforms was used as a dedicated research platform.

As we had the availability over questionnaire data including questions on, e.g., symptoms, earlier SARS-CoV-2 infections and eating, smoking, drinking and tooth brushing habits within one hour before testing, these data were used to further analyze the discordant results of the OP/NP swabs and saliva samples. We hypothesized that specific eating/drinking or smoking habits may have influenced the saliva samples, although some studies showed no difference resulting in a negative test result, but this was not confirmed by our data [16]. When the questionnaire data from participants with positive saliva samples but a negative OP/NP swab were analyzed, no significant associations were found, although it appeared that these participants had more often experienced a previous SARS-CoV-2 infection and showed fewer complaints at the moment of testing, which may indicate a more effective immune response to the current infection compared to participants with concordant test results. Furthermore, the time-to-positivity (TTP) data within the discordant RT-TMA results showed that the TTP in the saliva positive samples from participants with a negative OP/NP swab was significantly larger (indicating a lower viral load) compared to the TTP in the concordant samples and in the OP/NP swabs from the participants with a negative saliva sample. This finding is in line with previous observations that indicate saliva as a reservoir in which SARS-CoV-2 may longer persist than in the nasopharynx [17]. On the other hand, evidence exists that ACE2 expression was present at detectable levels in salivary glands, indicating a salivary gland infection instead of a reservoir [18,19].

In the saliva samples, we observed 17 invalid results (9.1%) for the RT-TMA assay. This can be explained by the different diluting protocols that were used during the study. For the first samples, we used 0.5 mL PCR-grade water. After the first run, we changed the dilution protocol from 0.5 to 2 mL and experienced no invalid results anymore. Hanson et al. also experienced invalid testing results with a 1:1 dilution factor but did not alter their protocol during the study [9]. The effect of the dilution factor can be seen in the concordant RT-TMA time-to-positivity results, which were marginally higher for saliva (median (IQR): 12.6 (3.4)) compared to OP/NP (median (IQR): 9.8 (3.4)).

The RT-TMA assay simultaneously produces results for SARS-CoV-2 and Influenza A/B. This can be very helpful in future testing policies where SARS-CoV-2 and influenza emerge side by side. Discrimination between both viruses is important as clinical presentations overlap, but infection control policies and public health implications differ. We found three co-infections with SARS-CoV-2 and influenza. Several studies showed increased mortality within these patients, stressing the importance of early detection and treatment [20].

Although saliva is a non-invasive collection method compared to OP/NP sampling, laboratory processing is more challenging as it is more labor intensive and complicated steps are involved. A pipetting and diluting step (in high viscosity/thick samples) is currently needed when different methods (such as drooling or spitting) and sample collection kits are used. In a high-throughput setting, this could lead to labor-intensive processing. One way to bypass this disadvantage is using a pipetting robot, but this will not fix the problem of high viscosity, thick, stringy samples. This issue was already described in earlier studies [21].

## 5. Conclusions

In conclusion, this study shows that the Hologic Panther platform Real-Time Transcription-Mediated Amplification assay (RT-TMA) yields a sensitivity for the detection of SARS-CoV-2 in saliva that is comparable to OP/NP swabs derived from participants presenting themselves at a public health testing facility with minimal or mild symptoms. In addition, the RT-TMA assay also produces results for influenza and a viral load indication based on the time to positivity. Future studies must focus on the interpretation of the time to positivity in relation to the cycle threshold values. Saliva is a safe and non-invasive sampling method in situations where OP/NP sampling is not possible.

## Figures and Tables

**Table 1:** Age distribution and percentage SARS-CoV-2 positive of the study population compared to the public health service testing population.

Age Groups	Positive Public Health Testing	Number Tested Public Health Testing	% Pos Public Health Testing	Positive Study Population	Number Tested Study Population	% Pos Study Population
<12 year	507	794	63.9%	0	0	-
12–17 year	575	934	61.6%	2	3	66.7%
18–29 year	2202	3580	61.5%	18	38	47.4%
30–39 year	1870	2749	68.0%	19	33	57.6%
40–49 year	1544	2274	67.9%	28	38	73.7%
50–59 year	1652	2517	65.6%	28	46	60.9%
60–69 year	1090	1587	68.7%	16	20	80.0%
70–79 year	669	937	71.4%	6	7	85.7%
>79 year	174	249	69.9%	1	2	50.0%
Total	10,283	15,621	65.8%	118	187	63.1%

**Table 2:** Comparison of characteristics between TMA positive and negative persons.

		TMA Neg	TMA Pos	Total	OR (Univariable)
Age	Median (IQR)	37.0 (26.0 to 50.5)	48.0 (34.0 to 57.0)	46.0 (31.5 to 56.0)	1.03 (1.01–1.05, *p* = 0.015)
Time since symptom onset	Median (IQR)	1.0 (1.0 to 2.0)	1.0 (1.0 to 2.0)	1.0 (1.0 to 2.0)	0.98 (0.76–1.29, *p* = 0.905)
Number of days earlier infection	Median (IQR)	156.0 (63.8 to 386.8)	327.5 (124.2 to 456.2)	279.5 (103.8 to 438.2)	1.00 (1.00–1.01, *p* = 0.181)
Earlier infection	No	35 (68.6)	114 (83.8)	149 (79.7)	-
	Yes	16 (31.4)	22 (16.2)	38 (20.3)	0.42 (0.20–0.90, *p* = 0.024)
Sex	Female	29 (56.9)	74 (54.4)	103 (55.1)	-
	Male	22 (43.1)	62 (45.6)	84 (44.9)	1.10 (0.58–2.13, *p* = 0.764)
Cold	No	16 (31.4)	41 (30.1)	57 (30.5)	-
	Yes	35 (68.6)	95 (69.9)	130 (69.5)	1.06 (0.52–2.10, *p* = 0.871)
Sore throat	No	19 (37.3)	46 (33.8)	65 (34.8)	-
	Yes	32 (62.7)	90 (66.2)	122 (65.2)	1.16 (0.59–2.26, *p* = 0.661)
Cough	No	34 (66.7)	71 (52.2)	105 (56.1)	-
	Yes	17 (33.3)	65 (47.8)	82 (43.9)	1.83 (0.95–3.65, *p* = 0.078)
Headache	No	34 (66.7)	71 (52.2)	105 (56.1)	-
	Yes	17 (33.3)	65 (47.8)	82 (43.9)	1.83 (0.95–3.65, *p* = 0.078)
Myalgia	No	44 (86.3)	104 (76.5)	148 (79.1)	-
	Yes	7 (13.7)	32 (23.5)	39 (20.9)	1.93 (0.83–5.07, *p* = 0.147)
Short of breath	No	47 (92.2)	118 (86.8)	165 (88.2)	-
	Yes	4 (7.8)	18 (13.2)	22 (11.8)	1.79 (0.63–6.45, *p* = 0.314)
Stomach ache	No	46 (90.2)	131 (96.3)	177 (94.7)	-
	Yes	5 (9.8)	5 (3.7)	10 (5.3)	0.35 (0.09–1.31, *p* = 0.110)
Fever	No	42 (82.4)	115 (84.6)	157 (84.0)	-
	Yes	9 (17.6)	21 (15.4)	30 (16.0)	0.85 (0.37–2.09, *p* = 0.715)
Loss of smell and taste	No	48 (94.1)	129 (94.9)	177 (94.7)	-
	Yes	3 (5.9)	7 (5.1)	10 (5.3)	0.87 (0.23–4.15, *p* = 0.842)
Diarrhea	No	50 (98.0)	132 (97.1)	182 (97.3)	-
	Yes	1 (2.0)	4 (2.9)	5 (2.7)	1.52 (0.22–30.02, *p* = 0.713)
Skin rash	No	51 (100.0)	135 (99.3)	186 (99.5)	-
	Yes	0 (0.0)	1 (0.7)	1 (0.5)	-
Eat something	No	27 (52.9)	71 (52.2)	98 (52.4)	-
	Yes	24 (47.1)	65 (47.8)	89 (47.6)	1.03 (0.54–1.97, *p* = 0.929)
Drink something	No	15 (29.4)	43 (31.6)	58 (31.0)	-
	Yes	36 (70.6)	93 (68.4)	129 (69.0)	0.90 (0.44–1.80, *p* = 0.772)
Brushed teeth	No	29 (56.9)	88 (64.7)	117 (62.6)	-
	Yes	22 (43.1)	48 (35.3)	70 (37.4)	0.72 (0.37–1.39, *p* = 0.325)
Smoked	No	44 (86.3)	120 (88.2)	164 (87.7)	-
	Yes	7 (13.7)	16 (11.8)	23 (12.3)	0.84 (0.33–2.30, *p* = 0.716)
Chewing gum	No	44 (86.3)	120 (88.2)	164 (87.7)	-
	Yes	7 (13.7)	16 (11.8)	23 (12.3)	0.84 (0.33–2.30, *p* = 0.716)

## Data Availability

The data underlying this article will be shared on reasonable request to the corresponding author.
